# Testing a Biobehavioral Model of Food Insecurity and Chronic Disease in Hispanic Older Adolescents

**DOI:** 10.3390/nu15041027

**Published:** 2023-02-18

**Authors:** Diana Rancourt, Faith A. Heeren, Michelle Cardel

**Affiliations:** 1 Department of Psychology, University of South Florida, Tampa, FL 33620, USA; 2 Department of Health Outcomes and Biomedical Informatics, University of Florida, Gainesville, FL 32610, USA; 3 WW International, Inc., New York, NY 10010, USA

**Keywords:** food insecurity, adolescent, Hispanic or Latino, depression, feeding and eating disorders, cardiovascular diseases

## Abstract

The biobehavioral model of food insecurity and chronic disease posits that stress perpetuates the cycle of food insecurity and chronic disease, in part, through changes in eating behaviors and weight gain. The current study conducted a preliminary test of the biobehavioral model in a sample of Hispanic older adolescents. It was hypothesized that older adolescents experiencing food insecurity would report greater depressive symptoms, which would be associated with more disordered eating, which would be associated with worse cardiometabolic indicators. Hispanic older adolescents (*N* = 113; 60% female; 15–21 years with mean age of 19.1; BMI_mean_ = 24.4) completed self-report baseline measures of food insecurity, depression, and disordered eating behaviors as part of a larger experimental study. Anthropometrics and body composition, blood pressure, heart rate, and resting metabolic rate were objectively measured. Hypotheses were tested using structural equation modeling. Experiencing food insecurity was associated with more disordered eating (b = 2.20, *p* = 0.032). Greater depressive symptoms were associated with more disordered eating (b = 0.28, *p* = 0.025) and worse cardiometabolic indicators (b = 0.15, *p* = 0.017). The full biobehavioral model, however, was not supported. Findings underscore the complex interaction of social and psychological functioning and physical health.

## 1. Introduction

Food insecurity, defined as the lack of reliable access to a sufficient quantity of affordable food [1], has been a key issue in the domain of economic and instability in the Healthy People 2020 Social Determinants of Health topic area. Food insecurity is associated with weight gain and having overweight or obesity [2,3,4], increased mortality [5], and high health care use and costs [6]. The associations between food insecurity and poor physical outcomes are complex and may result from a myriad of factors [5] including disordered eating behaviors [7,8]. Physical outcomes may also be exacerbated by psychological mechanisms associated with food insecurity. This study conducted a preliminary test of a biobehavioral model of food insecurity and chronic disease among Hispanic older adolescents.

The biobehavioral model of food insecurity and chronic disease posits that stress perpetuates the cycle of food insecurity and chronic disease, in part, through changes in eating behaviors and weight gain [9]. Stress impacts this cycle behaviorally (increased risk of disordered eating behaviors, e.g., binge eating, dietary restriction [10]), and biologically (e.g., physiological changes that drive excess calories consumption [11] and/or increase likelihood of weight gain [10,12]). Consistent with this model, food insecurity is associated with disordered eating behavior including binge eating and restrictive behaviors [2,4,13,14,15] and psychological distress. Among individuals who experience food insecurity, dietary restriction is often used as a method to make available food last longer [7], which may lead to overeating and binge eating behaviors that increase the risk for weight gain [16,17].

Weight gain is a well-established risk factor for a number of chronic illnesses and associated mortality. For example, a higher weight is associated with increased risk for cardiovascular disease [18], cancer [19], and diabetes [20]. Obesity during adolescence contributes to risk of cardiovascular disease in adulthood [21], and a higher body mass index (>25 kg/m^2^) in adulthood is associated with increased mortality risk associated with cancer, cardiovascular disease, and blood endocrine disorders [22]. Understanding the factors that contribute to weight gain during adolescence has important implications for contributing to long-term health and longevity.

Psychological distress is associated with disordered eating behaviors and increased risk for weight gain [10,23,24,25,26]. Physiologically, both stress and disordered eating may cause biological changes that increase the risk for weight gain and the development of overweight or obesity [10,27,28]. For example, lower perceived socioeconomic status is associated with increases in active ghrelin and lower fullness and satiety [11]. This suggests that the experience of food insecurity alone may impact hunger regulation and appetite in a way that could increase the risk for weight gain and chronic disease. Furthermore, using dietary restriction as a strategy to stretch food availability may biologically increase the risk for weight gain by physiologically signaling that there is insufficient food available, leading the body to compensate by increasing fat storage and risk for weight gain [12]. Individuals with food insecurity who report high stress may be a particularly vulnerable group for disordered eating behavior and excess weight gain, putting them at greater risk for chronic disease and increased medical costs over time.

A challenge to empirically testing the biobehavioral model of food insecurity and chronic disease is the measurement of chronic stress. The term “stress” is unspecific and has been used to reflect affective and physiological responses to a wide range of experiences including public speaking and having a lower socioeconomic status [29]. In the context of the biobehavioral model of food insecurity, the concept of stress is intended to describe the cumulative psychological and physiological effects of food insecurity, which increase the risk for chronic disease. However, this description poses challenges to measuring these effects, especially among older adolescents. Stress measures designed for use with children and adolescents lack standardization and may conflate stressors and symptoms of psychopathology [30]. Depressive symptoms, however, may be a useful proxy for older adolescents’ experience of chronic stress. Adolescence is a developmental period during which there is heightened stress-induced hormonal responses during puberty-related changes in the reactivity of the hypothalamic–pituitary–adrenal (HPA) axis [31,32]. Stress contributes to atypical HPA functioning [33], which is associated with increased risk for depression among adolescents [33,34]. The current study used depressive symptoms as a proxy for chronic stress among Hispanic older adolescents.

Hispanic adolescents are a high-risk group for overweight/obesity and may be particularly susceptible to the processes outlined in the biobehavioral model of food insecurity and chronic disease. Hispanic adolescents have the greatest prevalence of obesity in the United States compared to youth of other backgrounds [35], and adolescent obesity increases the risk for cardiometabolic disease [36]. Furthermore, Hispanic youth experiencing food insecurity experience worse health outcomes compared to peers of other backgrounds including more depressive symptoms and higher weight status [37], and are at particular risk for disordered eating [38]. It was hypothesized that among Hispanic older adolescents, food insecurity would be associated with worse cardiometabolic functioning via more depressive symptoms and disordered eating behaviors.

## 2. Materials and Methods

### 2.1. Participants

This study was a secondary analysis of data from a randomized controlled trial investigating the effects of perceived social status and food insecurity on the eating behaviors of Hispanic older adolescents [39]. Participants were 133 Hispanic older adolescents (60.2% female) aged 15–21 years (*M* = 19.1, SD = 1.3) with an average body mass index (BMI) of 24.44 (SD = 4.11). Participants were recruited for the primary study via flyers and participant referral. Inclusion criteria were self-identification as a Hispanic adolescent, BMI between 18.5 and 40 kg/m^2^, and being born in the United States. Individuals were excluded if they had never played Monopoly© (Hasbro, Inc., Pawtucket, RI, USA), reported 1, 2, 9, or 10 on the community subjective social status scale during screening (for the purposes of successful experimental manipulation of social status in the primary study) [40], used tobacco, were pregnant, or had dietary restrictions, gained or lost ≥10 lb/4.5 kg in the previous six months, severe clinical depression, uncontrolled psychiatric disease (e.g., untreated anxiety disorder), known substance abuse, an eating disorder, any major health condition, or medication use known to affect body composition, appetite, metabolism, or cardiac function.

### 2.2. Procedures

The current study used data from the pre-experiment baseline portion of the larger study that experimentally manipulated social status to examine its effects on acute eating behavior [39]. Only pre-experimental baseline study methods will be described here. Interested individuals completed a telephone screening conducted by a research assistant. Eligible participants were instructed to fast for 12 h and avoid strenuous exercise for 24 h prior to participating in the study. Signed informed consent/assent was obtained from all participants involved in the study after fasting status was confirmed. Anthropometrics were measured including height, weight, and waist circumference. Participants rested for 10 min prior to the measurement of blood pressure and heart rate, followed by resting metabolic rate and percent body fat. Participants then reported feelings of stress, powerfulness, and hunger rated on a visual analogue scale; consumed a standardized breakfast; had heart rate and blood pressure re-measured; completed the visual analogue scales a second time; provided specimens for the assessment of salivary cortisol; and completed a series of questionnaires, a subset of which were used in the current study. Participants were masked to the main aim of the study, and, upon completion, participants were informed of the true study aim via email. Participants aged 18 years and older were compensated with a $25 USD gift card. Minor participants (i.e., younger than 18 years) chose between volunteer hours or a $25 USD gift card.

### 2.3. Measures

#### 2.3.1. Anthropometrics and Body Composition

Participants were weighed to the nearest 0.1 kg on a digital scale (Health-O-Meter 2600 KL Wheelchair Scale, Sunbeam Products, Boca Raton, FL, USA). Height was recorded to the nearest 0.1 cm using a wall-mounted stadiometer (Holtain Limited, Croswell Crymych, United Kingdom). BMI was calculated from the measured height and weight using the formula kg/m^2^. Percent body fat was assessed using BodPod (Cosmed, Concord, CA, USA). Waist circumference was measured against the skin with a tape measure wrapped around the umbilicus to the nearest 0.5 cm (1/4 inch).

#### 2.3.2. Blood Pressure and Heart Rate

After the anthropometrics were assessed, the participants rested for 10 min prior to providing their blood pressure and heart rate measurements [41].

#### 2.3.3. Resting Metabolic Rate

Resting metabolic rate (RMR) was assessed using the Parvo Medics TrueOne 2400 machine by having participants lie in an active resting state for 30 min. During the 30 min period, the amount of oxygen inhaled and carbon dioxide exhaled was recorded every 30 s. The first and last 5-min increments were discarded, and the remaining measurements were averaged to determine the RMR [42]. The machine was calibrated daily before use according to the manufacturer’s protocols.

#### 2.3.4. Food Insecurity

Food insecurity was measured using the two-item clinical screener for food insecurity [43]. Food insecurity was dichotomized to reflect 0 = food secure, 1 = food insecure.

#### 2.3.5. Depressive Symptoms

The 21-item Beck Depression Inventory-II (BDI-II) measures the severity of self-reported depression and is appropriate for use with individuals aged 13–80 years [44]. Items are rated on a scale from 0 to 3 and sum scored, with higher scores indicating more depressive symptoms and greater severity of symptoms. Scores were interpreted as follows: 0–13 minimal depressive symptoms; 14–19 mild symptoms; 20–28 moderate symptoms; and ≥29 severe symptoms. A score of ≥21 indicated optimally differentiated adolescent inpatients with and without a depressive disorder [45].

#### 2.3.6. Disordered Eating

The 26-item Eating Attitudes Test-26 (EAT-26) [46] measures a range of self-reported disordered eating behaviors. Items are rated on a scale of 1 = Always to 6 = Never and recoded on a scale of 0 to 3, with scores of 3 reflecting the most disordered response option. A sum score of ≥20 reflects a high risk for an eating disorder [46].

### 2.4. Data Analysis

Descriptive statistics were generated using SPSS v27. Missing data were minimal and listwise deletion was employed for the descriptive analyses. Maximum likelihood estimation was used for all structural equation modeling. All continuous indicators except for waist circumference (skewness = 2.02, kurtosis = 8.67) and the EAT-26 (skewness = 2.03) met the item normality assumptions (i.e., items did not exceed skewness of an absolute value ≥2 and/or kurtosis of an absolute value of ≥7 [47]).

Prior to testing the hypotheses, a measurement model was tested to identify a latent cardiometabolic risk indicator variable in Mplus v8.3 with goemin (oblique) rotation and full maximum likelihood estimation with robust standard errors (MLR). The scree plot [48] and parallel analysis [49] were consulted to identify the number of factors. Adequate standardized item loadings were defined as a primary loading of ≥0.40. Item cross-loading was defined as a secondary loading of ≥0.30 or a gap of ≥0.20 between the primary and secondary loading [50]. The comparative fit index (CFI) values ≥0.95, root mean square error of approximation (RMSEA) values ≤0.06, and standardized root mean square residual (SRMR) values of ≤0.08 were used to indicate a good model fit [51]. Acceptable fit may be demonstrated with RMSEA values <0.08, and SRMR values <0.09 [52,53], while RMSEA values between 0.08 and 0.10 indicate a marginal fit [52].

Subsequent to developing the cardiometabolic risk latent variable, a cross-sectional indirect effect model with MLR was estimated using Mplus v8.3. This model specified food insecurity as the independent variable and depressive symptoms and disordered eating as serial indirect effects. The dependent variable was the latent cardiometabolic risk variable. Indirect effects were tested using the MODEL INDIRECT command.

## 3. Results

### 3.1. Sample Characteristics and Descriptive Statistics

Table 1 presents the descriptive statistics. Thirty-four participants (25.6%) reported experiencing food insecurity, four participants (3.0%) were at high risk for an eating disorder, and two participants (1.5%) were at high risk for a depressive disorder. Thirty-five participants (26.3%) met the criteria for overweight (25.0 ≤ BMI < 30) and 13 (9.8%) met the criteria for obesity (BMI ≥ 30). Disordered eating was significantly and positively correlated with experiencing food insecurity and resting metabolic rate. Depressive symptoms were significantly and positively correlated with waist circumference, percent body fat, and diastolic blood pressure (see Table 2).

### 3.2. Cardiometabolic Risk Indicator Measurement Model

An initial exploratory factor analysis included BMI, systolic blood pressure, diastolic blood pressure, heart rate, RMR, percent body fat, and waist circumference as indicators of the latent cardiometabolic risk variable. The scree plot and parallel analysis indicated a single factor; however, the fit statistics were poor, Χ^2^(14) = 80.49, *p* < 0.001, CFI = 0.47, RMSEA = 0.19, SRMR = 0.12. All indicators had acceptable standardized item loadings except heart rate (0.12). A subsequent EFA excluding heart rate similarly indicated a unifactor model, but this model also demonstrated poor model fit, Χ^2^(9) = 62.83, *p* < 0.001, CFI = 0.46, RMSEA = 0.21, SRMR = 0.11. In this model, the standardized loading of diastolic blood pressure fell below the threshold (0.39). Removal of this indicator improved the model fit, Χ^2^(5) = 22.68, *p* < 0.001, CFI = 0.72, RMSEA = 0.16, SRMR = 0.10, though the fit remained suboptimal. RMR did not load sufficiently strongly in this subsequent model (0.38) and was removed. A final EFA demonstrated acceptable model fit, Χ^2^(2) = 5.62, *p* = 0.06, CFI = 0.91, RMSEA = 0.12, SRMR = 0.06, and all indicators (BMI, systolic blood pressure, percent body fat, waist circumference) met the predetermined standardized loading thresholds. This latent variable was included in subsequent hypothesis testing as the dependent variable, with higher scores indicating greater cardiometabolic risk.

### 3.3. Hypothesis Testing

**Figure 1:**
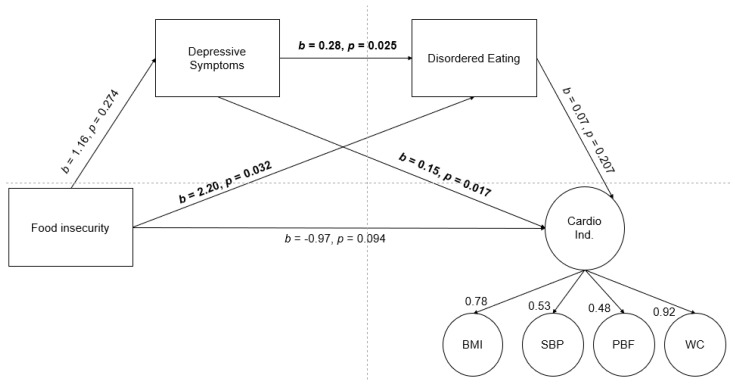
Test of the biobehavioral model of food insecurity with a latent cardiometabolic risk indicator variable as the outcome. Unstandardized effects presented for the structural equation model. Standardized factor loading estimates for the latent variable. Cardio Ind. = cardiometabolic risk indicators. BMI = body mass index. SBP = systolic blood pressure. PBF = percent body fat. WC = waist circumference. Bolded estimates are significant at *p* < 0.05.

The initial indirect effect model demonstrated suboptimal, fit, Χ^2^(11) = 23.98, *p* = 0.013, CFI = 0.872, RMSEA = 0.094, SRMR = 0.051. Modification indices indicated that allowing BMI and percent body fat to correlate would significantly improve the model fit. The final indirect effect model demonstrated a good model fit, Χ^2^(10) = 12.88, *p* = 0.231, CFI = 0.972, RMSEA = 0.047, SRMR = 0.029. The serial indirect effect was not significant, b = 0.02, *p* = 0.447 (see Figure 1); however, there were several significant direct effects. Compared to teens without food insecurity, teens living with food insecurity reported more disordered eating behaviors, b = 2.20, *p* = 0.032. Teens reporting greater depressive symptoms reported more disordered eating behaviors, b = 0.28, *p* = 0.025 as well as greater cardiometabolic risk, b = 0.15, *p* = 0.017.

### 3.4. Sensitivity Analyses

The indirect effect model was re-estimated using a state report of stress instead of the BDI as a measure of stress. The model demonstrated a marginally acceptable fit, Χ^2^(10) = 20.48, *p* = 0.025, CFI = 0.891, RMSEA = 0.089, SRMR = 0.038. The same pattern of findings was observed, with one exception. In this model, more disordered eating was associated with greater cardiometabolic risk (*b* = 0.12, *p* = 0.035).

## 4. Discussion

As no indirect effect of food insecurity on cardiometabolic functioning was observed, the present study did not provide support for the biobehavioral model of food insecurity and chronic disease [9] in a sample of Hispanic older adolescents. Direct effects were consistent with the extant literature. Having food insecurity and greater depressive symptoms each were independently associated with disordered eating, which is a risk factor for weight gain and developing overweight/obesity [16,17], and greater depressive symptoms were associated with greater cardiometabolic risk [54,55,56,57]. The findings underscore the importance of considering mental health in the prevention of chronic disease in Hispanic older adolescents.

There are a number of possibilities for why the current study did not provide support for the biobehavioral model of food insecurity [9]. First, the biobehavioral model describes the long-term impact of experiencing food insecurity and chronic stress on chronic disease, whereas this study was cross-sectional in nature. It is possible that a multi-year longitudinal study could demonstrate associations more consistent with the model. Second, the study included older adolescents. The impact of chronic stress on health might not yet be measurable in this age group. Third, chronic stress was operationalized as depressive symptoms. Future investigations of the biobehavioral model of food insecurity would benefit from a multimodal approach to measuring stress that includes depressive symptoms, perceived stress, a negative affect, and objective measures (e.g., hair sample analysis). Due to the exclusion criteria of the primary study from which these data originated, participating Hispanic older adolescents reported low levels of depressive symptoms and disordered eating behaviors, which may have attenuated the results and obfuscated the true indirect effects. Nonetheless, the results provide support for several important aspects of the model and outline opportunities for future work to test the tenets of the biobehavioral model of food insecurity more comprehensively.

The present study provides additional evidence of the association between food insecurity and disordered eating behaviors, but not depressive symptoms, among Hispanic older adolescents. Food insecurity is associated with disordered eating behaviors among adolescents [2,4,13,14,15] and poor psychological functioning including internalizing symptoms and behavioral problems in children and adolescents [58,59,60]. There was no association between experiencing food insecurity and depressive symptoms in the present study, which may be due to the aforementioned floor effects. Only 25% of the sample reported experiencing food insecurity, which is lower than some reports among the Hispanic/Latino populations (e.g., 42%) [37]. This lower rate of food insecurity, paired with the low rates of depressive symptoms, may have attenuated the association between these constructs. An alternative explanation is that the participants had not been experiencing food insecurity for enough time to see a demonstrable impact on their psychosocial functioning; however, no data were collected on how long households had been experiencing food insecurity. It will be useful for future work to explore the association between the duration of food insecurity and psychosocial functioning to clarify whether there is a generalizable inflexion point after which significant decreases in functioning are observed.

Emerging work links depressive symptoms and greater cardiovascular risk factors in youth [54,55,56,57]. The present study similarly demonstrated a significant association between depressive symptoms and cardiometabolic risk. Although the cross-sectional nature of the data precludes temporal or causal conclusions, the findings signal that depressive symptoms may increase the risk for cardiometabolic risk factors in Hispanic older adolescents. Ensuring adequate access to mental health treatment is broadly important and may also be a useful preventive intervention to decrease cardiometabolic risk in older adolescents. Unfortunately, families experiencing food insecurity are unlikely to have the resources for mental health services. Developing accessible and low-cost options for mental health support and treatment (e.g., low-cost telehealth, school-based services) has the potential to have far-reaching impacts for both the mental and physical health of Hispanic youth, adolescents, and young adults.

The study has several strengths including a strong theoretical basis, a variety of measured cardiometabolic risk factors, a large sample of Hispanic older adolescents—a typically underrepresented population that experiences health inequities—and a relatively sex-balanced sample. Our findings should be considered in light of some limitations. First, the data were cross-sectional, limiting the ability to understand how the constructs of interest influence health over time. Future research should test the biobehavioral model using longitudinal data, ideally over decades. Second, depressive symptoms were used as a proxy for stress. Although the experimental study from which the data were obtained included self-report and salivary cortisol measures of stress, these measures were intended to reflect the changes in the state levels of stress. Future work should measure a variety of types of stress including life, interpersonal, and academic stress to comprehensively investigate the biobehavioral model. Third, disordered eating behaviors were measured from the perspective of risk for an eating disorder. Individuals experiencing food insecurity engage in disordered eating behaviors for different motivations than individuals with shape and weight concerns. Future research would benefit from assessing the motivations for disordered eating behaviors among individuals with food insecurity, as this may have implications for the prevention and treatment of these behaviors among food insecure individuals. Fourth, the participants were relatively healthy; the study for which individuals were recruited expressly excluded individuals with clinically significant levels of depressive symptoms and/or disordered eating. This may have attenuated the findings and limits the generalizability to community samples that include individuals struggling with psychopathology. Nonetheless, the participants reported a range of depressive symptoms and disordered eating, and it is likely that the findings would be stronger with greater variability—including the severity—of depressive and disordered eating symptoms. Fifth, the sample size was not large enough to test the hypotheses separately by sex. The rates of depressive symptoms and disordered eating varies by sex among the adolescents and young adults [61,62]. Furthermore, body fat varies across males and females during and post-puberty [63]. Future research would benefit from recruiting a sufficiently large sample to test for differences across sex and pubertal status.

## 5. Conclusions

While the present study did not provide support for the biobehavioral model of food insecurity and chronic disease, our findings replicated extant work for several tenets of the model. Among Hispanic older adolescents, those experiencing food insecurity reported more disordered eating, and more depressive symptoms were associated with both more disordered eating and worse cardiometabolic indicators, which are associated with increased risk for chronic disease. Overall, the results underscore the importance of considering the effects of social conditions on the health behaviors and the impact of the psychological functioning on physical health.

## Figures and Tables

**Table 1:** Descriptive statistics of the variables of interest (*N* = 133).

Variable	Mean/Frequency (Standard Deviation)
Age	19.14 (1.29)
Sex at birth (female)	60.2%
Student status (student)	86.5%
Body mass index	24.44 (4.11)
Underweight	4.5%
Healthy weight	59.4%
Overweight	26.3%
Obesity	9.8%
Waist circumference (cm; *n* = 132)	33.16 (5.01)
Percent body fat (*n* = 131)	25.71% (6.92)
Systolic blood pressure	112.42 (10.91)
Diastolic blood pressure	69.37 (7.54)
Heart rate	67.39 (11.17)
Resting metabolic rate (*n* = 119)	1176.93 (250.41)
Food insecurity	25.6%
Disordered eating	4.65 (4.85)
Depressive symptoms	5.87 (5.23)
Minimal	91.0%
Mild	7.5%
Moderate	1.5%
Severe *	0.0%

Note. * The primary study from which these data originated excluded participants who met the criteria for a depressive disorder.

**Table 2:** Correlations among the variables of interest (*N* = 133).

Variable	1	2	3	4	5	6	7	8	9	10	11	12
1. Age	--											
2. Sex	−0.01	--										
3. BMI	0.08	−0.18 *	--									
4. WC	0.11	−0.30 **	0.71 **	--								
5. %BF	−0.01	0.37 **	0.66 **	0.46 **	--							
6. SBP	0.04	−0.57 **	0.43 **	0.49 **	0.13	--						
7. DBP	0.06	−0.07	0.31 **	0.31 **	0.31 **	0.60 **	--					
8. HR	0.01	0.33 **	0.07	0.04	0.28 **	0.12	0.35 **	--				
9. RMR	0.04	−0.44 **	0.37 **	0.33 **	0.03	0.45 **	0.22 *	0.12	--			
10. FI	0.10	−0.02	−0.02	−0.10	−0.01	−0.03	−0.05	0.01	0.04	--		
11. DE	0.03	−0.05	0.15	0.13	0.06	0.07	−0.02	−0.01	0.19 *	0.23 **	--	
12. Dep	0.09	0.07	0.16	0.26 **	0.18 *	0.13	0.18 *	0.03	0.02	0.10	0.32	--

Note. Sex = indicated female sex at birth. BMI = body mass index. WC = waist circumference in inches (*n* = 132). %BF = percent body fat (*n* = 131). SBP = systolic blood pressure. DBP = diastolic blood pressure. HR = heart rate. RMR = resting metabolic rate (*n* = 119). FI = have food insecurity. DE = disordered eating. Dep = depressive symptoms. * = *p* < 0.05. ** = *p* < 0.01.

## Data Availability

The data presented in this study are available upon request from the last author.
